# Glycolysis-Based Genes Are Potential Biomarkers in Thyroid Cancer

**DOI:** 10.3389/fonc.2021.534838

**Published:** 2021-04-26

**Authors:** Feng Xu, Huan Xu, Zixiong Li, Yuanyuan Huang, Xiaoling Huang, Yangyi Li, Xiaohe Zheng, Yongsong Chen, Ling Lin

**Affiliations:** ^1^Department of Respiratory and Critical Care Medicine, The First Affiliated Hospital of Shantou University Medical College, Shantou, China; ^2^Clinical Research Center, The First Affiliated Hospital of Shantou University Medical College, Shantou, China; ^3^Department of Rheumatology, The First Affiliated Hospital of Shantou University Medical College, Shantou, China; ^4^Department of Thyroid and Breast Surgery, The First Affiliated Hospital of Shantou University Medical College, Shantou, China; ^5^Department of Endocrinology, The First Affiliated Hospital of Shantou University Medical College, Shantou, China

**Keywords:** tumor glycolysis, thyroid cancer, gene signature, TME, prognosis

## Abstract

While increased glycolysis has been identified as a cancer marker and attracted much attention in thyroid cancer (THCA), the prognostic role of it remains to be further elucidated. Here we aimed to determine a specific glycolysis-associated risk model to predict THCA patients' survival. We also explored the interaction between this signature and tumor immune microenvironment and performed drug screening to identify specific drugs targeting the glycolysis-associated signature. Six genes (CHST6, POM121C, PPFIA4, STC1, TGFBI, and FBP2) comprised the specific model, which was an independent prognostic indicator in THCA patients determined by univariate, LASSO and multivariate Cox regression analyses. The receiver operating characteristic (ROC) curve analysis confirmed the excellent clinical performance of the prognostic signature. According to the specific gene signature, patients were categorized into high- and low-risk subgroups. The high-risk group was characterized by decreased immune score and elevated tumor purity, as well as worser survival prognosis compared to the low-risk group. We also validated the expression of these genes in clinical samples and *in-vitro* experiments. Lastly, we identified potential drugs targeting the glycolysis-associated signature. The derived glycolysis-related signature is an independent prognostic biomarker for THCA patients and might be used as an efficacy of biomarker for drug-sensitivity prediction.

## Introduction

Thyroid cancer (THCA) is one of the most frequently diagnosed malignancies of the endocrine system worldwide, and this cancer incidence rate is still on the rise (1–3). The average annual incidence rate of THCA is more than 6%, which is the highest among all cancers (4). Although THCA is considered to be a curable disease after standard treatment, tumor recurrence, and distant metastasis result in unsatisfactory clinical results in a small proportion of patients. Thus, there is a real need to investigate novel and effective factors, which may predict THCA patient prognosis more accurately.

Warburg effect, also known as aerobic glycolysis, is a phenomenon whereby various types of cancer cells characterize by excessive conversion of glucose to lactate for their energy substrate regardless of oxygen levels (5). Growing evidence indicates that accelerated glycolysis in cancers influence the therapy outcome that most cancers show significant increases in glucose uptake when compared with adjacent normal tissue (6–8). Moreover, increased glycolysis has been reported to promote angiogenesis and invasive cancer growth (9). Lactate, produced by glycolytic tumor cells, plays crucial roles in the suppression of anticancer immune cells and then promotes the tumor recurrence following anticancer therapies (10). The high accumulation of lactate in tumor microenvironment (TME), which lowers extracellular pH to 6.0–6.5, blocks the function and proliferation rate of T cells (11). High concentrations of lactate in TME affects antitumor therapy, which leads to the suggestion that inhibiting glycolytic pathway, and therefore lactate production may provide an effective and potential strategy to enhance anticancer agents.

In our study, we established a glycolysis-related gene model, which may be a robust prognostic indicator for clinical use. In addition, by applying ESTIMATE algorithm, we gained insight into the interaction of glycolysis-related gene signature with TME. *In-vivo* and *in-vitro* experiments confirmed the influence of glycolysis-related gene on tumor growth. Finally, we discovered candidate compounds targeting the glycolysis-related gene signature through the publicly available drug sensitivity database.

## Materials and Methods

### Data Collection

All RNA-seq expression profile and the clinical data for THCA patients were obtained from the Cancer Genome Atlas (TCGA) database. Our study meets TCGA's publication guidelines. Glycolysis-associated gene sets were downloaded from publicly available gene set databases-Molecular Signatures Database v7.0, namely three different gene sets (KEGG_GLYCOLYSIS_GLUCONEOGENESIS, HALLMARK_GLYCOLYSIS, and REACTOME_GLYCOLYSIS). These gene sets are presented in the Supplementary Table 1.

### Construction of Glycolysis-Related Risk Model

We performed a univariate Cox regression analysis to consider the association between glycolysis-related gene expression level and THCA's over survival, and genes were identified significantly when the *p* < 0.05. After primary filtration, the least absolute shrinkage and selection operator (LASSO) logistic regression with ten-fold cross validation was conducted to reduce glycolysis-related genes for THCA patients by using R package “glmnet.” Finally, the glycolysis-related risk model was finally established by a multivariate Cox regression analysis to identify the prognostic value of specific gene signature as our study previously described (12). THCA patients were then divided into high- and low-risk groups through the median score as a cutoff. The Kaplan-Meier method was applied to evaluate the significant difference of overall survival using “survival” R package between high- and low-risk groups. The receiver operating characteristic (ROC) analysis was applied to estimate the sensitivity and specificity of the prediction model.

### The cBioPortal Analysis

The cBioPortal for cancer genomics provides visualization features and analyzes multidimensional cancer genomics data (13). We used the THCA (TCGA, Firehouse Legacy) dataset for genetic mutations of glycolysis-related genes. The genomic profiles were determined as mutations, mRNA expression Z scores (RNA-seq v.2 RSEM), putative copy number alterations from GISTIC, and protein expression Z scores (RPPA).

### Estimating Immune Microenvironment

To predict the proportion of immune score and tumor purity in the TME of each THCA patient, we applied the ESTIMATE algorithm to estimate the immune score in THCA patients from the TCGA cohort (14, 15). Based on the ESTIMATE score, tumor purity was acquired using a fitted formula as previous study described (15).

### Independence of the Glycolysis-Related Gene Model From Other Clinical Features

In order to explore whether the prognostic signature was independent of other clinical variables, univariate, and multivariate Cox analyses were carried out.

### Cell Culture

The Nyth-ori-3-1, BCPAP, and TPC-1 cell lines were obtained from Guangzhou JENNIO Biotech Technology (Guangzhou, China). Nyth-ori-3-1 and TPC-1 cells were cultured in RPMI 1640 (GIBCO, Invitrogen, Carlsbad CA, USA), supplemented with 10% fetal bovine serum (FBS) (GIBCO, Melbourne, Australia). BCPAP cells were cultured in Dulbecco's modified Eagle's medium (DMEM) (GIBCO) containing 10% FBS of Australia origin. All the cells were cultured at 37°C in 5% CO_2_.

### RNA Isolation and Quantitative Real-Time PCR Analysis

Total RNA of Nyth-ori-3-1, TPC-1, and BCPAP cells was extracted utilizing Trizol method and 500 ng total RNA was reversely transcribed into cDNA with “PrimeScript^TM^ RT reagent Kit with gDNA Eraser” (Takara, Japan). Quantitative real time-PCR (qRT-PCR) was perform using “SYBR Green Premix PCR Master Mix” (Takara, Japan) according to the manufacturer protocols. We calculated the relative mRNA expression markers utilizing the Ct method (2^−ΔΔCt^) after being normalized to β-actin. All reactions were carried out independently and repeated three times each time. A primer sequence of the six genes was used and is presented in the Supplementary Table 2.

### Immunohistochemistry

The tissue samples were obtained from THCA patients and nodular goiter patients after surgery in our hospital. In addition, the sections were created after the tissues were dehydrated and embedded. The Ethics Committee from our hospital approved all the procedures of our study. Formalin-fixed paraffin embedded (FFPE) sections were subjected to antigen retrieval using citrate buffer for 15 min at 100°C and incubated in anti-CHST6 (1:30 Lifespan), anti-FBP2 (1:50 Abcam), anti-PPFIA4 (1:300 Abcam), anti-TGFBI (1:100 Abcam), and anti-STC1 (1:300 Abcam) at 4°C overnight. The primary antibody was omitted for negative-control sections. Sections were washed and placed in a biotinylated secondary antibody. After washing, the biotinylated secondary antibody, avidin-biotin complex, and horseradish peroxidase were applied (all the reagents were made from MXB, CHINA). Peroxidase activity was visualized by using DAB staining, which were then counterstained with hematoxylin (16–18). The figures of Immunohistochemistry were captured using a Nikon-inverted research-grade microscope.

The expression localizations of the glycolysis-associated genes in THCA tissues are clarified in Supplementary Table 3. Then the expression levels of target proteins in tissue were examined by two independent pathologists blinded to the clinical characteristics of the patients according to proportion of cell staining (0 = 0%, 1 = ≤25%, 2 = 26–50%, 3 = 51–75%, 4 = >75% positive cells) and the staining intensity (0 = no staining, 1 = weak, 2 =moderate, 3 = strong). A final score was calculated by multiplying the above two scores (19, 20). Protein expression was considered high if the final score was >6 points and low if the final score was 6 points or less. The specific scores of immunohistochemistry are clarified in Supplementary Table 4.

### Ethics Statement

Ethics approval for this project was obtained from the First Affiliated Hospital of Shantou University Medical College Ethics committee (No. B-2020-217).

### Therapeutic Response Prediction

With the R package “pRRophetic,” the drug-response prediction was estimated based on the half maximal inhibitory concentration (IC50) of each THCA patient on the Genomics of Drug Sensitivity in Cancer (GDSC) website (21).

## Results

### Identification of Glycolysis-Related Genes Significantly Correlated With Patients' Survival

**Figure 1:**
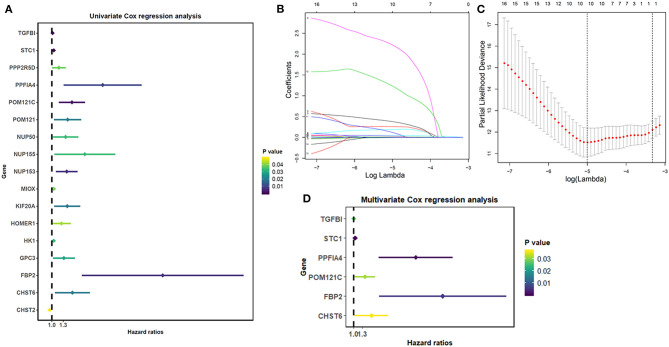
Identification of glycolysis-related genes significantly correlated with patients' survival. **(A)** The Univariate Cox analysis of glycolysis-related genes. **(B)** LASSO coefficient profiles of the glycolysis-related genes. **(C)** Plots of the cross-validation error rates. **(D)** Multivariate Cox analysis of glycolysis-related genes.

A univariate Cox regression was used to explore the interaction of the glycolysis-related genes with the overall survival of THCA patients and determined 17 survival-related genes in THCA patients when the *p* < 0.05 (Figure 1A). Then, a LASSO-penalized Cox analysis was developed to narrow the genes, which were selected over 900 times a total of 1,000 repetitions (Figures 1B,C). As a consequence, 10 genes were identified. In addition, a stepwise multivariate Cox regression analysis was performed, and six glycolysis-related genes were finally selected to construct the prognostic gene signature (Figure 1D).

### Construction of the Prognostic Glycolytic Gene Signature in TCGA

**Figure 2:**
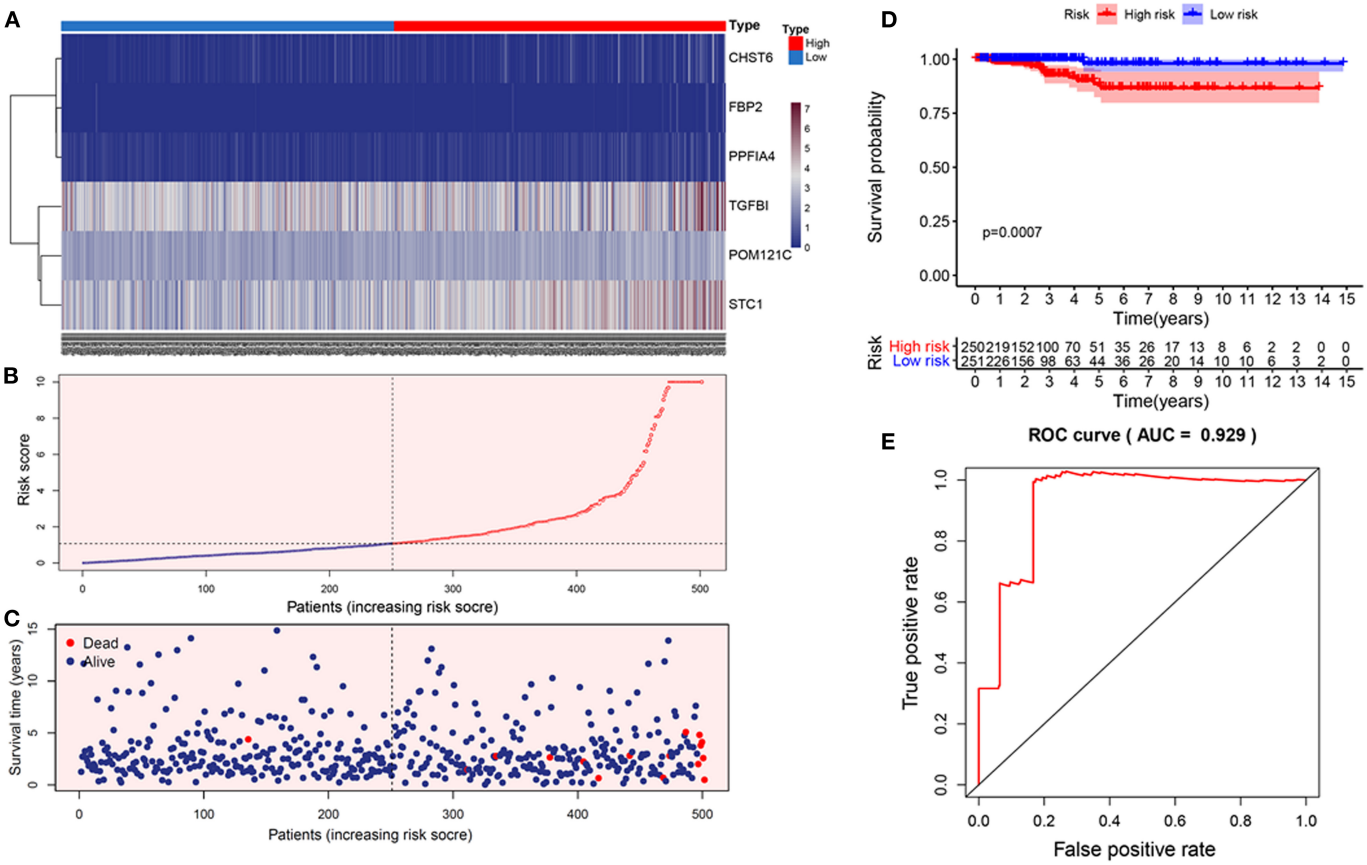
Construction of the prognostic glycolytic gene signature in TCGA. **(A)** Heatmap of six-gene expression profiles between the high- and low-risk groups. **(B)** The distribution of the glycolysis-based risk score. **(C)** Vital statuses of patients between the high- and low-risk groups. **(D)** Kaplan-Meier survival curves of the relative overall survival of high- and low-risk patients. **(E)** ROC curve analysis.

The risk score for predicting prognostic value was calculated using the formula: risk score = (0.0149 × TGFBI expression level) + (0.0517 × STC1 expression level) + (1.866 × PPFIA4 expression level) + (0.345 × POM121C expression level) + (0.542 × CHST6 expression level) + (2.672 × FBP2 expression level). We calculated the risk score for each THCA patient according to this formula and categorized the patients into high- or low-risk groups (Figures 2A–C). Kaplan-Meier analysis showed that high-risk patients had significantly worse overall survival than low-risk patients (*p* = 0.0007; Figure 2D). The prognostic capacity of the six-gene signature was assessed by calculating the area under the curve (AUC) of a time-dependent ROC curve (Figure 2E). The higher AUC demonstrated the better model performance for THCA-specific survival. The AUC of ROC analysis for the six-gene signature was 0.929, implying excellent performance for survival prediction.

### Glycolysis-Related Gene Mutations and the Correlation With Gene Expression in THCA Patients

**Figure 3:**
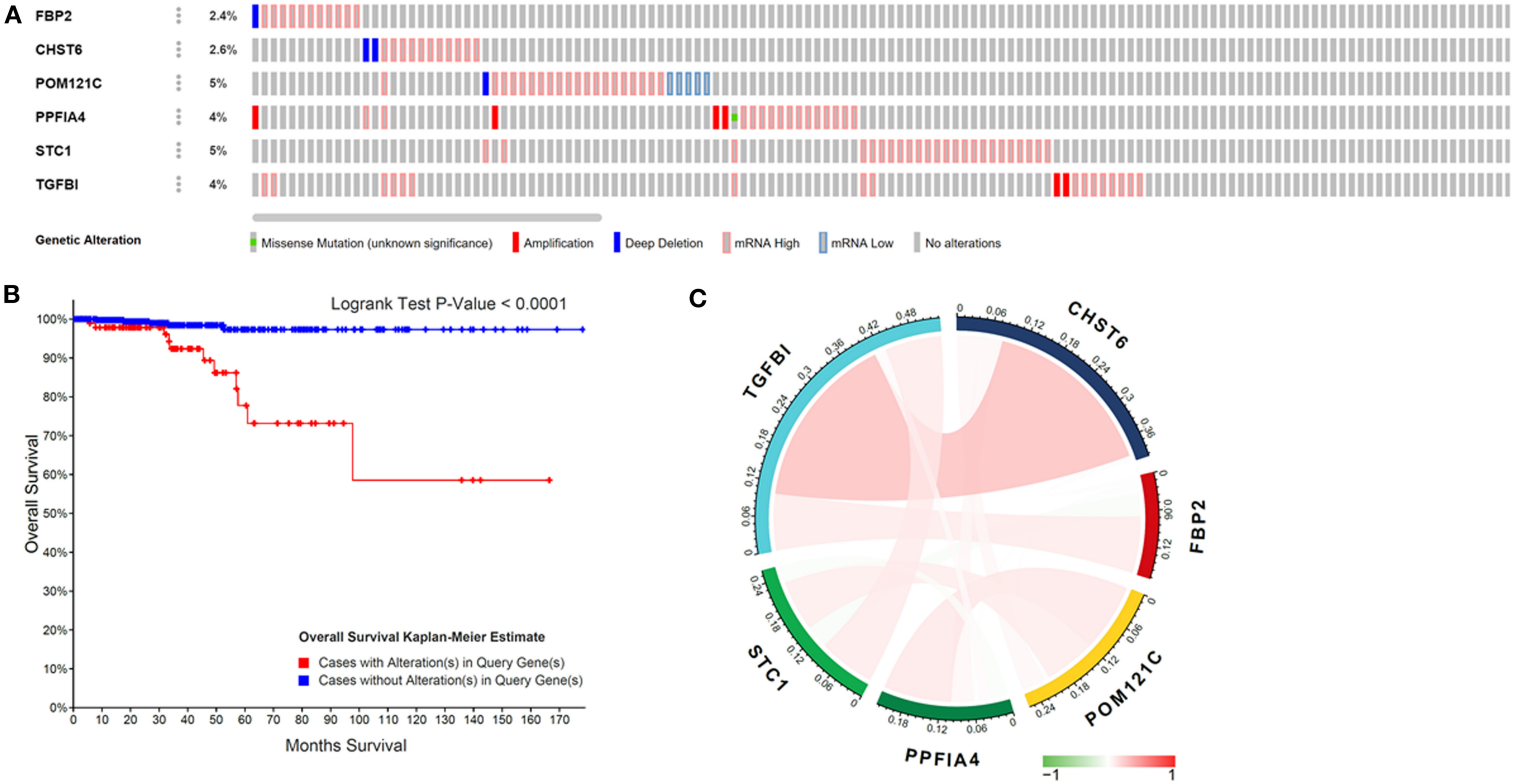
Glycolysis-related gene mutations and the correlation with gene expression in THCA patients. **(A)** Mutations of six genes in patients with THCA. **(B)** Kaplan–Meier survival curve for THCA patients stratified by the six-gene mutations. **(C)** Pearson correlation of six genes.

Genetic mutations of six genes were analyzed through cBioPortal online tool for THCA patients. Six genes were altered in 98 samples of 516 patients with THCA (19%) (Figure 3A). According to the relationship between the six-gene status and disease prognosis indicated that patients with these gene mutations showed poorer prognosis (Figure 3B), indicating that the glycolysis-related gene mutation may contribute to THCA progression. A Pearson correlation analysis was performed using gene expression data of six glycolysis-related genes collected from TCGA for THCA patients (Figure 3C). The results found out low correlations between each glycolysis-related gene, suggesting that these six genes were independent of each other.

### Association Between Tumor Immune Microenvironment and Gene Signature-Based Subsets in THCA

**Figure 4:**
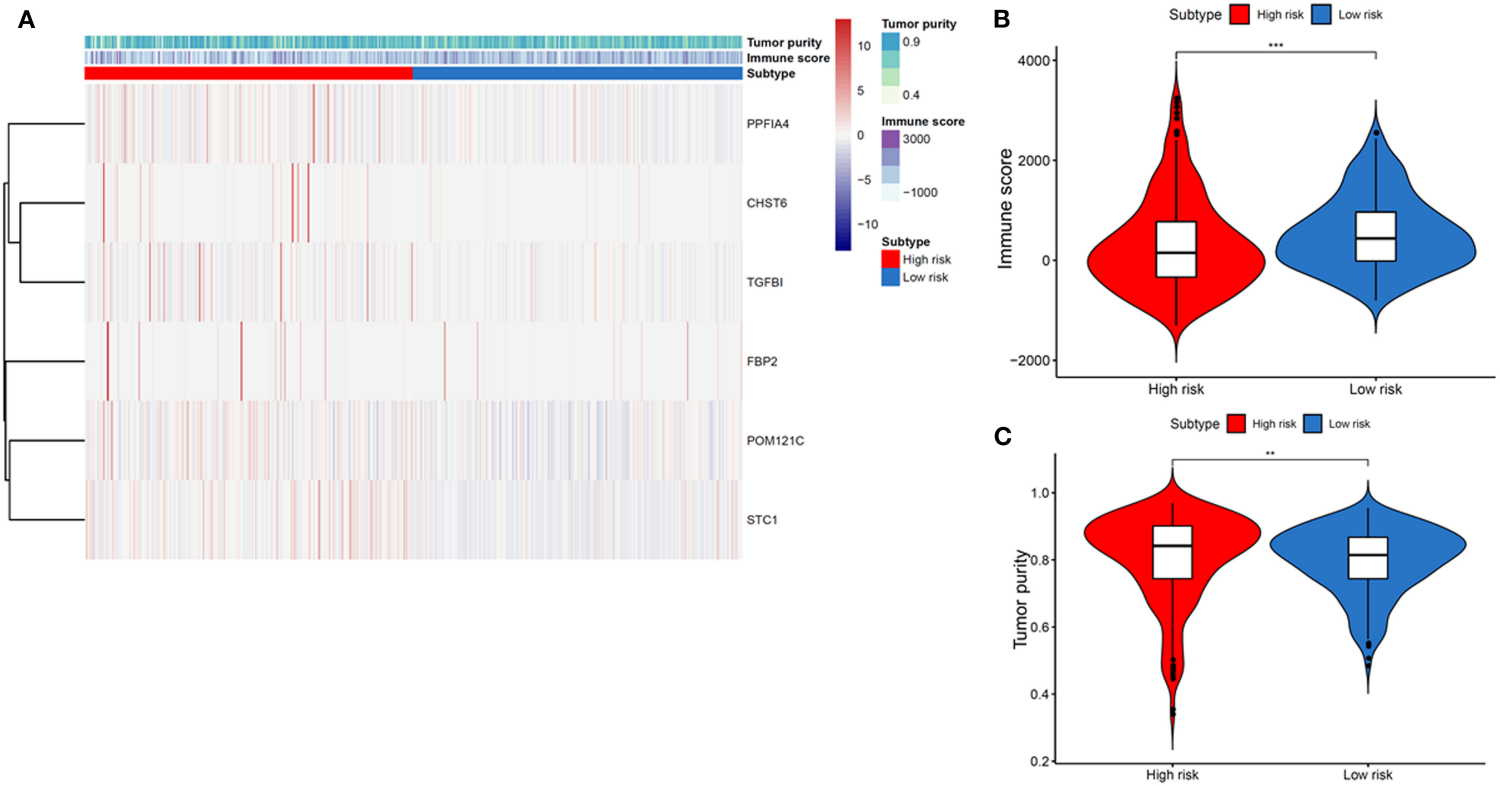
Association between tumor immune microenvironment and gene signature-based subsets in THCA. **(A)** Heatmap indicating the relationship of THCA subtypes with the expression of tumor immune microenvironment. **(B)** Immune score in THCA subtypes. **(C)** Tumor purity in THCA subtypes.

We then explored the TME differences in high- and low-risk THCA patients. As a result, TME were significantly different in high- and low-risk THCA patients (Figure 4A). On the basis of the ESTIMATE algorithm, the immune score in low-risk group was higher than those in high-risk group (Figure 4B). In addition, we compared the tumor purity of the two groups, and found the opposite trend (Figure 4C). These results showed that the glycolysis-related genes had significantly and negatively correlations with immune microenvironment, and the poor prognosis of the high-risk group was partly due to the immunosuppressive microenvironment.

### Associations Between the Glycolysis-Related Gene Signature and Clinical Properties

**Figure 5:**
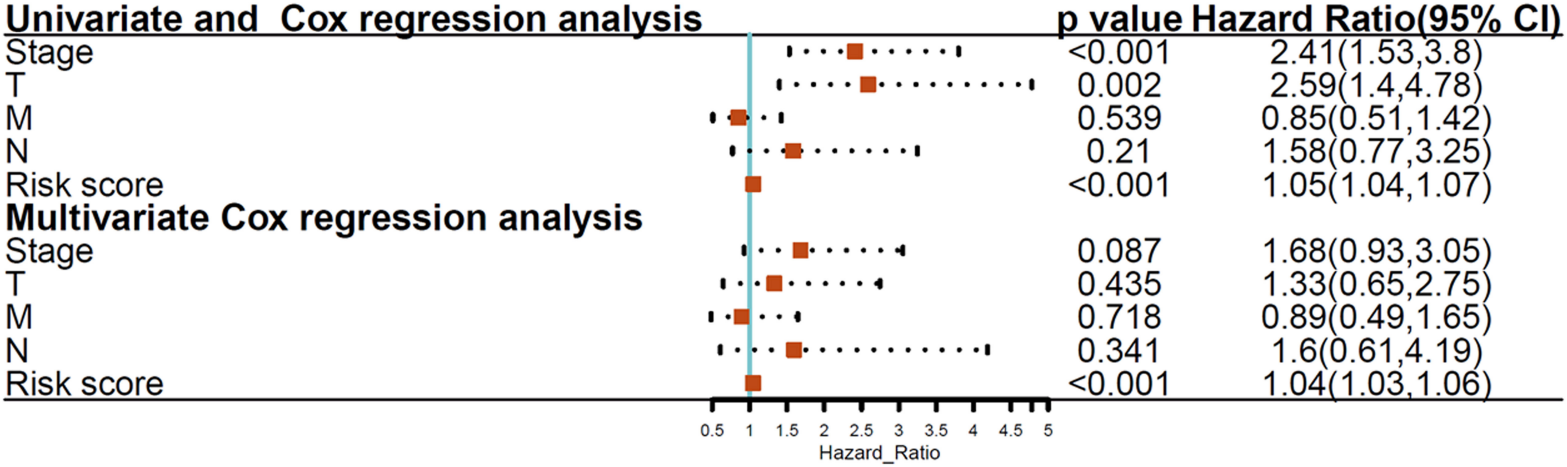
Univariate and multivariate Cox regression analyses between the glycolysis-related gene model and clinical features with overall survival.

We then elucidated whether the glycolysis-related gene model was an independent marker compared to clinical properties. Univariate Cox regression analysis revealed the T stage, TNM stage, and risk score were significantly associated with THCA patient prognosis, and multivariate Cox regression analysis documented that the glycolysis-related gene signature showed a remarkable prognostic value when compared with other clinical properties (*p* < 0.001; Figure 5).

### Measurement of Glycolysis-Related Genes at mRNA and Protein Levels in Cell Lines and Clinical Samples

**Figure 6:**
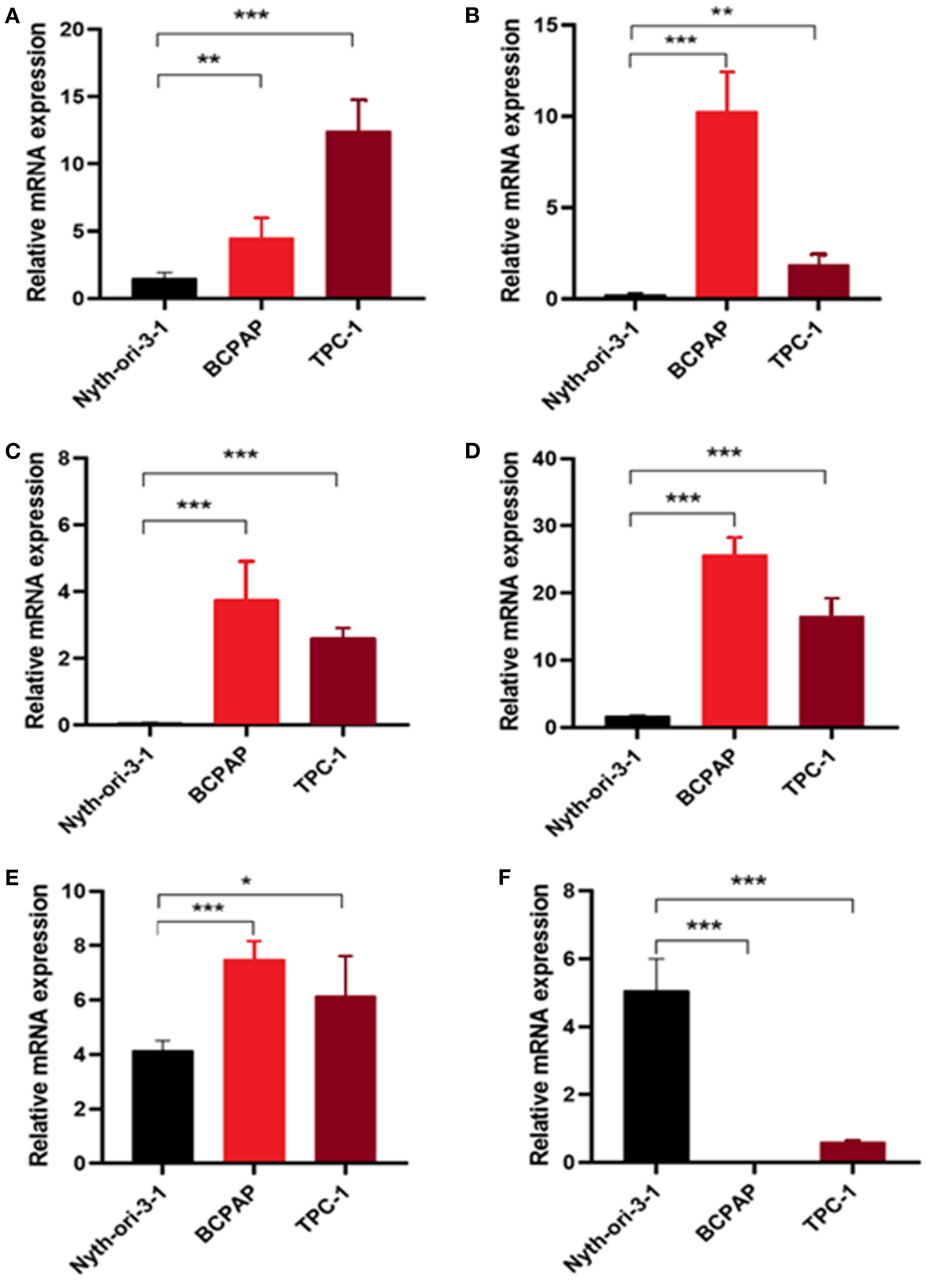
Measurement of glycolysis-related genes at mRNA levels in cell lines. Relative mRNA levels of **(A)** CHST6, **(B)** FBP2, **(C)** PPFIA4, **(D)** POM121C, **(E)** TGFBI, and **(F)** STC1 in thyroid cancer cells (BCPAP and TPC-1) and thyroid cells (Nyth-ori-3-1). *p < 0.05, **p < 0.01, and ***p < 0.001.

**Figure 7:**
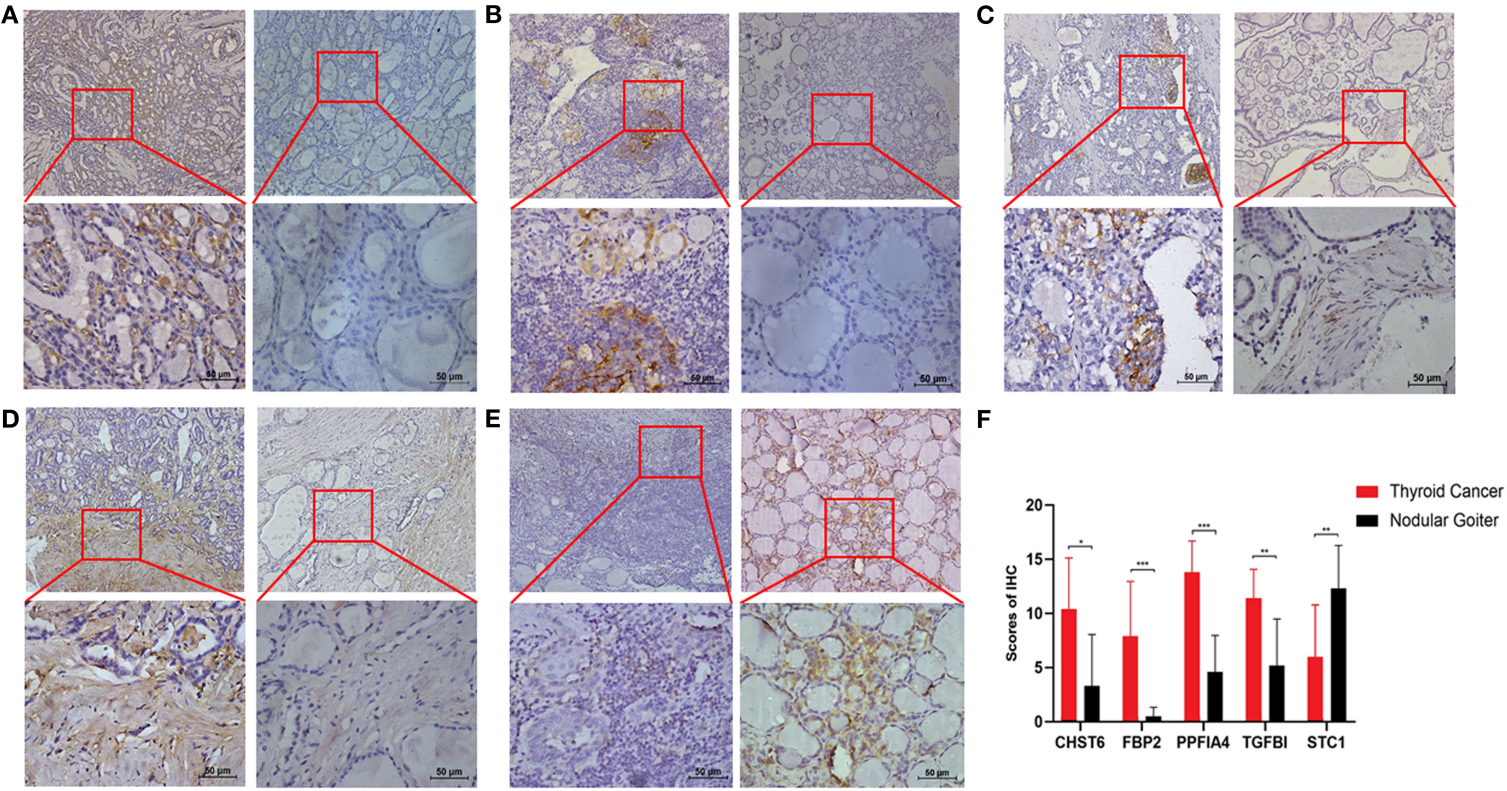
Measurement of glycolysis-related genes at protein levels in clinical samples. Representative immunohistochemical staining images of **(A)** CHST6, **(B)** FBP2, **(C)** PPFIA4, **(D)** TGFBI, and **(E)** STC1 in human thyroid cancer sections (left line) and nodular goiter sections (right line). **(F)** Protein expression scores in human thyroid cancer sections and nodular goiter sections. *p < 0.05, **p < 0.01, and ***p < 0.001.

**Figure 8:**
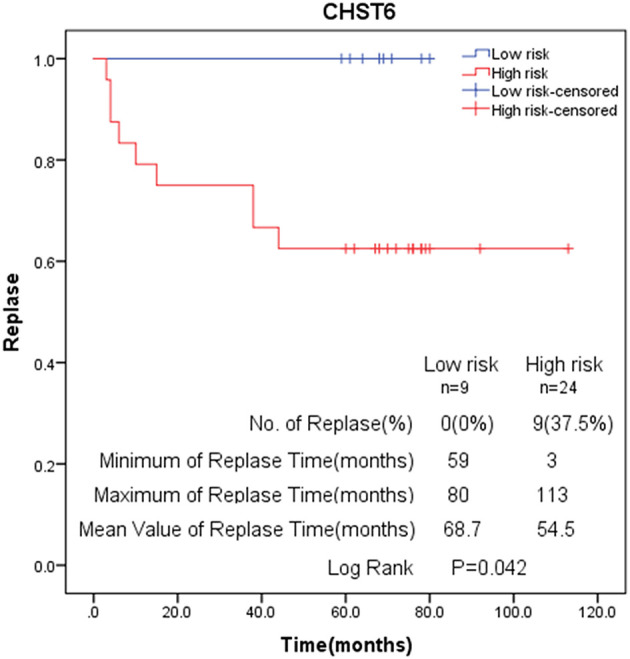
Kaplan–Meier analysis of recurrence rate according to CHST6 expression level.

To further validate the results, qRT-PCR was applied to analysis the relative mRNA expressions of six glycolysis-related genes in THCA cells (BCPAP, TPC-1) and normal thyroid cells (Nyth-ori-3-1). The results showed that THCA cell lines exhibited relative higher mRNA levels of CHST6, FBP2, PPFIA4, POM121C, and TGFBI, but a lower mRNA level of STC1 than normal thyroid cells (Figures 6A–F). In addition, immunohistochemistry analysis was also conducted to determine CHST6, FBP2, PPFIA4, TGFBI, and STC1 protein expression levels in THCA patients. According to the immunostaining, we could observe the similar results (Figures 7A–E). CHST6, FBP2, PPFIA4, and TGFBI proteins were all upregulated in THCA tissues compared with nodular goiter tissues. On the other hand, the result of STC1 was opposite (Figure 7F). In order to verify the specific efficacy in predicting recurrence of these genes, we collected patients who were diagnosed with THCA in our hospital in 2011–2015 and did follow-up surveys until November in 2020 to know their prognosis. According to the results of the immunohistochemistry, we divided those patients into a low-risk group and high-risk group and analyzed the recurrence rates of patients with THCA. The result showed that the recurrence rate in the high-risk group was higher than that in the low-risk group, according to the classification of the expression of CHST6 (Figure 8). In addition, the relationship between CHST6 and clinicopathologic factors of THCA patients is clarified in Supplementary Table 5.

### Analysis of GDSC Database Identifies Novel Candidate Compounds Targeting the Glycolysis-Related Gene Model

**Figure 9:**
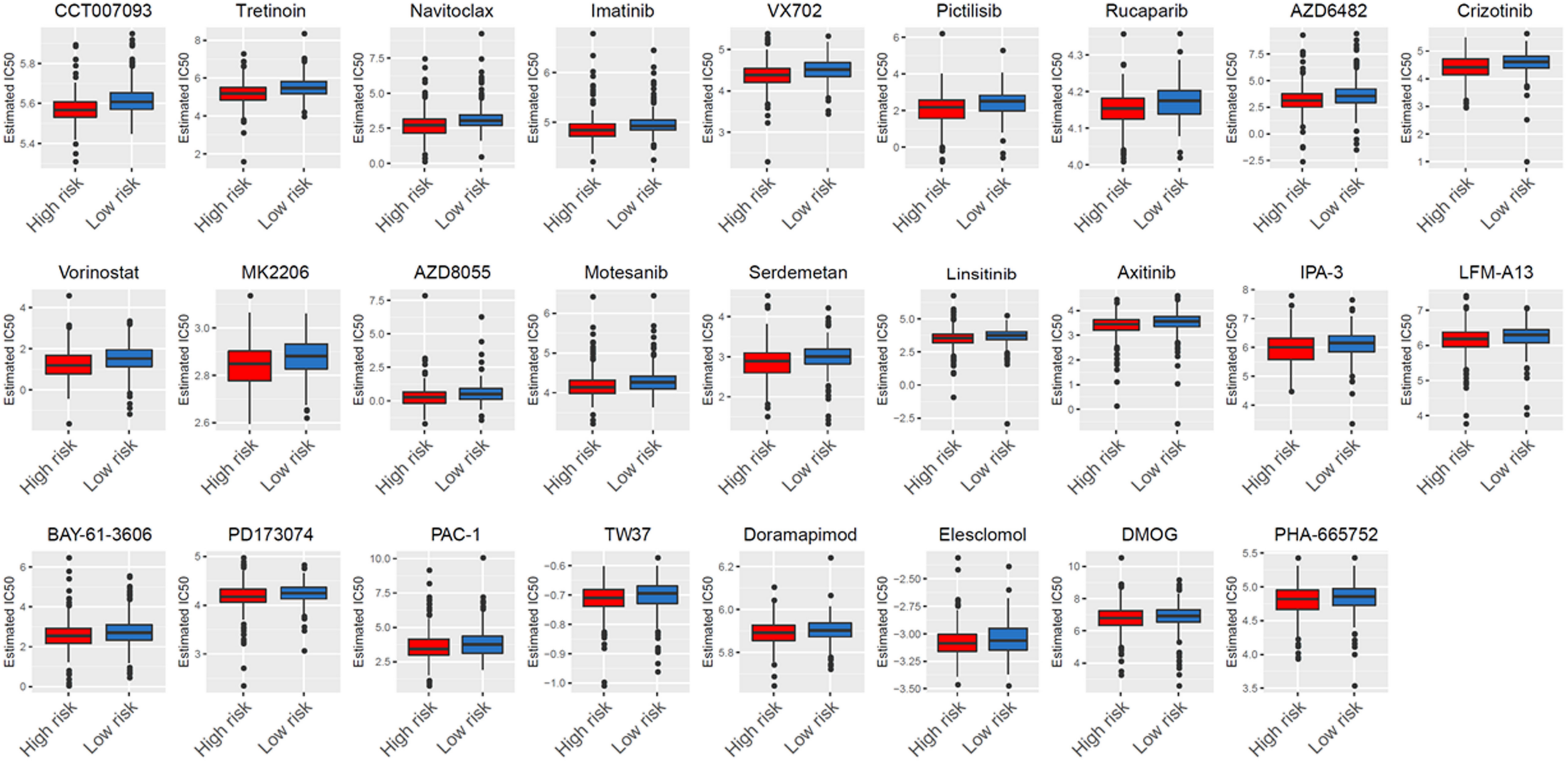
Analysis of GDSC database identifies novel candidate compounds targeting the glycolysis-related gene model.

After characterizing the key features of six glycolysis-related genes, we also explore potential compounds that capable of targeting the pathways linked to glycolysis on the basis of IC50 available in the GDSC database for each TCGA sample. It was excited that 26 chemo compounds were selected with significant differences in the estimated IC50 between high- and low-risk groups, and that the high-risk group was more sensitive to all of these drugs (Figure 9 and Table 1). These findings further suggested that the heterogeneity of glycolysis activation in THCA patients was a better model for predicting the therapeutic response.

**Table 1:** Screened drugs with selective sensitivity targeting glycolysis-associated signature.

**GDSC name**	***P*-Value**	**Targets**	**Target pathway**
CCT007093	<0.0001	PPM1D	Cell cycle
Tretinoin	<0.0001	Retinoic acid	Other
Navitoclax	<0.0001	BCL2, BCL-XL, BCL-W	Apoptosis regulation
Imatinib	<0.0001	ABL, KIT, PDGFR	Kinases
VX702	<0.0001	p38	JNK and p38 signaling
Pictilisib	<0.0001	PI3K	PI3K/MTOR signaling
Rucaparib	<0.0001	PARP1, PARP2	Genome integrity
AZD6482	<0.0001	PI3Kbeta	PI3K/MTOR signaling
Crizotinib	<0.0001	MET, ALK, ROS1	RTK signaling
Vorinostat	<0.0001	HDAC inhibitor Class I, IIa, IIb, IV	Chromatin histone acetylation
MK2206	<0.0001	AKT1, AKT2	PI3K/MTOR signaling
AZD8055	<0.0001	MTORC1, MTORC2	PI3K/MTOR signaling
Motesanib	<0.0001	VEGFR, RET, KIT, PDGFR	RTK signaling
Serdemetan	<0.0001	MDM2	p53 pathway
Linsitinib	<0.0001	IGF1R	IGF1R signaling
Axitinib	<0.0001	PDGFR, KIT, VEGFR	RTK signaling
IPA-3	0.0002	PAK1	Cytoskeleton
LFM-A13	0.0003	BTK	Kinases
BAY-61-3606	0.0005	SYK	Kinases
PD173074	0.0006	FGFR1, FGFR3	RTK signaling
PAC-1	0.0006	Procaspase-3, Procaspase-7	Apoptosis regulation
TW37	0.0025	BCL2, BCL-XL, MCL1	Apoptosis regulation
Doramapimod	0.0116	p38, JNK2	JNK and p38 signaling
Elesclomol	0.012	HSP90	Protein stability and degradation
DMOG	0.0248	HIF-PH	Metabolism
PHA-665752	0.0311	MET	RTK signaling

According to GDSC database analysis, five drugs (Crizotinib, Axitinib, Motesanib, PHA-665752, and PD173074) shared the RTK signaling pathway, four drugs (Pictilisib, AZD6482, AZD8055, and MK2206) shared the PI3K/MTOR signaling pathway, three drugs (PAC-1, Navitoclax, and TW37) shared the apoptosis regulation pathway, three drugs (BAY-61-3606, Imatinib, and LFM-A13) shared the kinases pathway, and two drugs (VX702 and Doramapimod) shared the JNK and p38 signaling. We also observed DMOG as a metabolism inhibitor, Linsitinib as an IGF1R signaling inhibitor, Rucaparib as a genome integrity inhibitor, Vorinostat as a chromatin histone acetylation inhibitor, CCT007093 as a cell cycle inhibitor, Elesclomol as a protein stability and degradation inhibitor, and Serdemetan as a p53 pathway inhibitor.

## Discussion

Recently, studies on immune evasion and energy metabolism have attracted people's attention, and the emerging hallmarks of cancer have been discovered (22–24). Unlike normal cells, cancer cells rely mainly on glycolysis for producing ATP energy, even when in the presence of adequate levels of oxygen (25). Many researchers also have explored the glucose metabolism features of THCA (26, 27). Thus, targeting the glycolytic pathway may have the promising future to provide an effective target for THCA therapy. Our study has identified glycolysis-related genes providing a new prognostic biomarker and therapeutic target for THCA patients. The AUC of ROC curve of this prediction model was 0.929, revealing this gene signature has an excellent effect in predicting survival. THCA patients were categorized into high- and low-risk groups through a glycolytic risk-prognosis model, and the overall survival rate of high-risk patients was worse. Clinical analysis also showed that THCA patients with the six-gene mutation have a poorer survival prognosis. In addition, qRT-PCR and immunohistochemistry were also applied to confirm the differential expressions of these glycolysis-related genes between THCA patients and non-tumor patients. We also found the recurrence rate in the high-risk group was higher than that in the low-risk group, according to the classification of the expression of CHST6. Those results indicated that these glycolysis-related genes might play crucial roles in determining the prognosis of cancer patients with THCA.

According to glycolysis-related signature, the clinician could establish individualized treatment for THCA patients. Additionally, experimental evidence indicated the accumulation of extracellular lactate produced by glycolytic cancer cells was related to the inhibition of anticancer immune cells. For instance, the high concentration of lactate in TME affected T cells' proliferation and function through disturbing their intracellular pH (28). Tumor-derived lactate was an important factor regulating dendritic cell phenotype in a TME and might be related to the tumor avoidance mechanism (29). Moreover, lactate, increased arginase I (ARG1) expression in macrophages, inhibited proliferation and activation of T-cell (30). Natural killer (NK) cells could also be inhibited by lactate, hence allowing for cancer progression (10). According to these reasons, we hypothesized that different groups of patients may have different immune responses. As consequence, we reported a significant negative correlation between glycolytic activity (high-risk group) and immune activity (quantified by immune score and tumor purity). Thus, we may further support the immunosuppressive role of glycolysis in patients with THCA, and suppress glycolysis to improve the immune status to increase the survival of patients with THCA.

We documented that the clinical TNM stage, T stage, and risk score indicated significant association with overall survival of THCA patients. What's more, we confirmed that the six-gene signature indicated an indispensable relationship with survival compared with other clinical characteristics. In standard clinical practice, the pathologic stage is considered to be an important prognostic determinant of THCA. However, there are some differences in clinical outcomes differ among patients at the same stage, demonstrating the present staging systems are inadequate for effective prognosis, and the biological heterogeneity of patients with THCA cannot be fully reflected. Thus, it is vital to obtain novel biomarkers to use as prognostic and therapeutic factors. To our knowledge, this is the first glycolysis-associated gene model confirmed in THCA. Our model provides a new method for the evaluation of THCA patients and guides prognostic prediction and treatment decisions.

Finally, according to the GDSC database, high-risk THCA patients were found to be more sensitive to 22 compounds compared with low-risk THCA patients. Twenty-two compounds revealed 13 mechanisms shared by the above compounds. Among the 22 compounds, Crizotinib, Axitinib, PD173074, Motesanib, and PHA-665752 shared the RTK signaling pathway. The RTK signaling stimulated the accumulation of cellular metabolites, thereby increasing lactate excretion, which led to T cell activity inhibition (31). Pictilisib, AZD6482, MK2206, and Serdemetan shared the PI3K/MTOR signaling pathway. The PI3K oncogene has been reported to stimulate glycolysis and promote cancer growth in a variety of human cancers (32–35). The mTOR, a downstream effector of PI3K/Akt signaling, had two forms and both were involved in the regulation of glycolysis (36, 37). We also explore other approaches that may eventually contribute to the implementation of targeted glycolysis therapy.

Our research provides a new perspective for the study of THCA immune microenvironment. However, as our study was retrospective, our study needed to be validated by further prospective studies. In addition, most public database data included in the analysis were from patients in developed countries but data from developing countries were lacking.

In conclusion, our study identifies a six-gene model related to glycolysis, which could independently predict THCA patient prognosis. In addition, *in-vivo* and *in-vitro* experiments reveal that expression of glycolysis-related genes are associated with tumor growth, which may be helpful to provide new therapeutic target for THCA patients in the future. Our study also identifies several specific drugs targeting glycolysis for individualized treatment.

## Data Availability Statement

All datasets generated for this study are included in the article/Supplementary Material.

## Ethics Statement

The studies involving human participants and animal studies were reviewed and approved by the First Affiliated Hospital of Shantou University Medical College Ethics committee (Shantou, China).

## Author Contributions

FX did the original design of the study, analyzed data, and wrote the manuscript. HX, ZL, YH, XH, and YL collected clinical samples. HX performed the *in vivo* and *in vitro* experiments. FX, LL, and YC provided funding acquisition. LL, YC, and XZ supervised the research, analyzed data, and wrote the manuscript. All authors read and approved the final submitted manuscript.

## Conflict of Interest

The authors declare that the research was conducted in the absence of any commercial or financial relationships that could be construed as a potential conflict of interest.
