# Supplementary material for: Glycolysis-Based Genes Are Potential Biomarkers in Thyroid Cancer
Source: Front Oncol. 2021 Apr 26;11:534838. doi: 10.3389/fonc.2021.534838 (PMC8107473; doi:10.3389/fonc.2021.534838)
Supplement: Supplementary file 1 [file Table_1.DOCX]

**Supplemental Table 1. The glycolysis-related gene sets on the three different gene sets.**

| KEGG_GLYCOLYSIS_ GLUCONEOGENESIS | HALLMARK_GLYCOLYSIS | REACTOME_GLYCOLYSIS |
| --- | --- | --- |
| ACSS2 | PGK1 | AAAS |
| GCK | ALDOA | ADPGK |
| PGK2 | ENO1 | ALDOA |
| PGK1 | TPI1 | ALDOB |
| PDHB | PFKP | ALDOC |
| PDHA1 | ERO1A | BPGM |
| PDHA2 | ALDOB | ENO1 |
| PGM2 | VEGFA | ENO2 |
| TPI1 | MXI1 | ENO3 |
| ACSS1 | PKM | GAPDH |
| FBP1 | HK2 | GAPDHS |
| ADH1B | LDHA | GCK |
| HK2 | EXT1 | GCKR |
| ADH1C | SLC25A10 | GNPDA1 |
| HK1 | GUSB | GNPDA2 |
| HK3 | PFKFB1 | GPI |
| ADH4 | PGAM1 | HK1 |
| PGAM2 | PYGB | HK2 |
| ADH5 | AK4 | HK3 |
| PGAM1 | P4HA1 | NDC1 |
| ADH1A | PMM2 | NUP107 |
| ALDOC | FAM162A | NUP133 |
| ALDH7A1 | SDC1 | NUP153 |
| LDHAL6B | EGLN3 | NUP155 |
| PKLR | PC | NUP160 |
| LDHAL6A | B4GALT7 | NUP188 |
| ENO1 | FBP2 | NUP205 |
| PKM | IGFBP3 | NUP210 |
| PFKP | CHPF | NUP214 |
| BPGM | B3GAT3 | NUP35 |
| PCK2 | CHST12 | NUP37 |
| PCK1 | HS2ST1 | NUP43 |
| ALDH1B1 | MPI | NUP50 |
| ALDH2 | GNPDA1 | NUP54 |
| ALDH3A1 | AKR1A1 | NUP58 |
| AKR1A1 | PPFIA4 | NUP62 |
| FBP2 | B3GAT1 | NUP85 |
| PFKM | CHPF2 | NUP88 |
| PFKL | G6PD | NUP93 |
| LDHC | MDH2 | NUP98 |
| GAPDH | CHST6 | NUPL2 |
| ENO3 | AC010618.1 | PFKFB1 |
| ENO2 | PGAM2 | PFKFB2 |
| PGAM4 | CHST1 | PFKFB3 |
| ADH7 | GPC1 | PFKFB4 |
| ADH6 | TSTA3 | PFKL |
| LDHB | ALG1 | PFKM |
| ALDH1A3 | GFPT1 | PFKP |
| ALDH3B1 | PRPS1 | PGAM1 |
| ALDH3B2 | GOT1 | PGAM2 |
| ALDH9A1 | MDH1 | PGK1 |
| ALDH3A2 | SLC35A3 | PGK2 |
| GALM | GALK1 | PGM2L1 |
| ALDOA | EGFR | PGP |
| DLD | ANGPTL4 | PKLR |
| DLAT | CITED2 | PKM |
| ALDOB | PLOD2 | POM121 |
| G6PC2 | QSOX1 | POM121C |
| LDHA | ME2 | PPP2CA |
| G6PC | SPAG4 | PPP2CB |
| PGM1 | P4HA2 | PPP2R1A |
| GPI | GAPDHS | PPP2R1B |
|  | ENO2 | PPP2R5D |
|  | GOT2 | PRKACA |
|  | EXT2 | PRKACB |
|  | SLC25A13 | PRKACG |
|  | HMMR | RAE1 |
|  | PDK3 | RANBP2 |
|  | CXCR4 | SEC13 |
|  | GPC4 | SEH1L |
|  | ECD | TPI1 |
|  | GNE | TPR |
|  | B4GALT2 |  |
|  | FUT8 |  |
|  | MIOX |  |
|  | VCAN |  |
|  | GPC3 |  |
|  | B3GALT6 |  |
|  | HSPA5 |  |
|  | ME1 |  |
|  | ADORA2B |  |
|  | UGP2 |  |
|  | MIF |  |
|  | NANP |  |
|  | ZNF292 |  |
|  | STC2 |  |
|  | TPST1 |  |
|  | PGM2 |  |
|  | GYS1 |  |
|  | TKTL1 |  |
|  | TGFA |  |
|  | CHST2 |  |
|  | PHKA2 |  |
|  | STMN1 |  |
|  | GALE |  |
|  | MET |  |
|  | LCT |  |
|  | IRS2 |  |
|  | POLR3K |  |
|  | B4GALT1 |  |
|  | EFNA3 |  |
|  | LHX9 |  |
|  | KDELR3 |  |
|  | TALDO1 |  |
|  | DPYSL4 |  |
|  | VLDLR |  |
|  | CD44 |  |
|  | AGL |  |
|  | SOX9 |  |
|  | DDIT4 |  |
|  | IDUA |  |
|  | CASP6 |  |
|  | GLCE |  |
|  | COPB2 |  |
|  | DSC2 |  |
|  | HS6ST2 |  |
|  | CDK1 |  |
|  | PLOD1 |  |
|  | SDC2 |  |
|  | GMPPB |  |
|  | PAXIP1 |  |
|  | NSDHL |  |
|  | RARS |  |
|  | SLC16A3 |  |
|  | GLRX |  |
|  | SRD5A3 |  |
|  | SDC3 |  |
|  | HDLBP |  |
|  | COL5A1 |  |
|  | CLDN9 |  |
|  | TFF3 |  |
|  | STC1 |  |
|  | KIF20A |  |
|  | GYS2 |  |
|  | SLC37A4 |  |
|  | LHPP |  |
|  | SDHC |  |
|  | NASP |  |
|  | AURKA |  |
|  | B3GNT3 |  |
|  | ISG20 |  |
|  | LDHC |  |
|  | ARPP19 |  |
|  | CENPA |  |
|  | HOMER1 |  |
|  | BIK |  |
|  | CYB5A |  |
|  | HAX1 |  |
|  | COG2 |  |
|  | IL13RA1 |  |
|  | AC074143.1 |  |
|  | CLDN3 |  |
|  | AGRN |  |
|  | CLN6 |  |
|  | TXN |  |
|  | PAM |  |
|  | CAPN5 |  |
|  | PKP2 |  |
|  | ABCB6 |  |
|  | DCN |  |
|  | GMPPA |  |
|  | BPNT1 |  |
|  | ANG |  |
|  | GPR87 |  |
|  | GAL3ST1 |  |
|  | ALDH7A1 |  |
|  | NT5E |  |
|  | IDH1 |  |
|  | PYGL |  |
|  | NDUFV3 |  |
|  | NDST3 |  |
|  | PPP2CB |  |
|  | PSMC4 |  |
|  | TPBG |  |
|  | TGFBI |  |
|  | GALK2 |  |
|  | CTH |  |
|  | KIF2A |  |
|  | CACNA1H |  |
|  | ANKZF1 |  |
|  | SAP30 |  |
|  | RBCK1 |  |
|  | ELF3 |  |
|  | RPE |  |
|  | B4GALT4 |  |
|  | DEPDC1 |  |
|  | RRAGD |  |
|  | IER3 |  |
|  | ALDH9A1 |  |
|  | DLD |  |
|  | MERTK |  |
|  | GCLC |  |
|  | FKBP4 |  |
|  | SOD1 |  |
|  | MED24 |  |
|  | AK3 |  |
|  | XYLT2 |  |
|  | ARTN |  |
|  | PPIA |  |
|  | CHST4 |  |
