# Supplementary material for: Glycolysis-Based Genes Are Potential Biomarkers in Thyroid Cancer
Source: Front Oncol. 2021 Apr 26;11:534838. doi: 10.3389/fonc.2021.534838 (PMC8107473; doi:10.3389/fonc.2021.534838)
Supplement: Supplementary file 2 [file Table_2.DOCX]

**Supplemental Table 2 Primer sequences of six genes for Quantitative real time-PCR**

| Gene | Forward primer | Reverse primer |
| --- | --- | --- |
| CHST6 | 5‘-CCAACACTTGTGCTGAGGAA-3’ | 5‘-TAGCGCCTGCTACAACTGTG3’ |
| FBP2 | 5‘-TCAACATGGTCCAATCCTCCT-3’ | 5‘-CATCCAGTGGGTCAAAGCAGA-3’ |
| PPFIA4 | 5‘-CTCTGCGGATGTTGTCTCCC-3’ | 5‘-ATGCTGCCACTGGTTACACG-3’ |
| POM121C | 5‘-AGAGGCTCAAATGCTGTCCC-3’ | 5‘-GCCAGGTATAACACTCGCC-3’ |
| TGFBI | 5‘-CTTTGAGACCCTTCGGGCTG-3’ | 5‘-CGTGTACTGGCCGTTACCTT-3’ |
| STC1 | 5‘-ATCACATTCCAGCAGGCTTC-3’ | 5‘-CCTGAAGCCATCACTGAGGT-3’ |
| β-actin | 5‘-TGGCACCCAGCACAATGAA-3’ | 5‘-CTAAGTCATAGTCCGCCTAGAAGCA-3’ |
