# Supplementary material for: Glycolysis-Based Genes Are Potential Biomarkers in Thyroid Cancer
Source: Front Oncol. 2021 Apr 26;11:534838. doi: 10.3389/fonc.2021.534838 (PMC8107473; doi:10.3389/fonc.2021.534838)
Supplement: Supplementary file 3 [file Table_3.DOCX]

**Supplemental Table 3 Cellular localization in Immunohistochemistry**

| **Gene** | **localization** |
| --- | --- |
| CHST6 | Golgi apparatus membrane, Cytoplasm |
| FBP2 | Cell junction, Cytoplasm, Nucleus |
| PPFIA4 | Cytoplasm, Cell surface |
| TGFBI | Secreted > extracellular space > extracellular matrix |
| STC1 | Secreted |
