# Supplementary material for: Glycolysis-Based Genes Are Potential Biomarkers in Thyroid Cancer
Source: Front Oncol. 2021 Apr 26;11:534838. doi: 10.3389/fonc.2021.534838 (PMC8107473; doi:10.3389/fonc.2021.534838)
Supplement: Supplementary file 4 [file Table_4.DOCX]

**Supplemental Table 4 The Result of Immunohistochemistry**

|  |  | Low risk(n=) | High risk(n=) | Total(n=) | Score  (mean value) | P value |
| --- | --- | --- | --- | --- | --- | --- |
| CHST6 | Thyroid Cancer | 2 | 8 | 10 | 7 | 0.035 |
|  | Nodular Goiter | 8 | 2 | 10 | 3 |  |
|  |  |  |  |  |  |  |
| FBP2 | Thyroid Cancer | 5 | 5 | 10 | 6.9 | 0.000 |
|  | Nodular Goiter | 10 | 0 | 10 | 0.5 |  |
|  |  |  |  |  |  |  |
| PPFIA4 | Thyroid Cancer | 1 | 9 | 10 | 11 | 0.000 |
|  | Nodular Goiter | 9 | 1 | 10 | 3.8 |  |
|  |  |  |  |  |  |  |
| TGFBI | Thyroid Cancer | 0 | 10 | 10 | 8.9 | 0.002 |
|  | Nodular Goiter | 3 | 7 | 10 | 4.4 |  |
|  |  |  |  |  |  |  |
| STC1 | Thyroid Cancer | 6 | 4 | 10 | 4.8 | 0.002 |
|  | Nodular Goiter | 1 | 9 | 10 | 10.2 |  |
