# Supplementary material for: Glycolysis-Based Genes Are Potential Biomarkers in Thyroid Cancer
Source: Front Oncol. 2021 Apr 26;11:534838. doi: 10.3389/fonc.2021.534838 (PMC8107473; doi:10.3389/fonc.2021.534838)
Supplement: Supplementary file 5 [file Table_5.DOCX]

**Supplemental Table 5 Relationship between CHST6 and clinicopathologic factors of THCA patients**

| Variables | Case (n = 33) | CHST6 level | | P value |
| --- | --- | --- | --- | --- |
|  |  | High(n=24) | Low(n=9) |  |
| Gender |  |  |  | 0.174 |
| Male | 3 | 1 | 2 |  |
| Female | 30 | 23 | 7 |  |
| Age |  |  |  | 1.000 |
| ＜60 | 28 | 20 | 8 |  |
| ≥60 | 5 | 4 | 1 |  |
| Tumor size |  |  |  | 0.033 |
| ≤ 3 cm | 20 | 12 | 8 |  |
| > 3 cm | 9 | 9 | 0 |  |
| Not available | 4 | 3 | 1 |  |
| Local invasion |  |  |  | 0.540 |
| T1 + T2 | 26 | 18 | 8 |  |
| T3 + T4 | 3 | 3 | 0 |  |
| Not available | 4 | 3 | 1 |  |
| Lymphatic invasion |  |  |  | 0.635 |
| N0 | 22 | 15 | 7 |  |
| N1 + N2 | 7 | 6 | 1 |  |
| Not available | 4 | 3 | 1 |  |
| Remote metastasis |  |  |  | / |
| M0 | 29 | 21 | 8 |  |
| M1 | 0 | 0 | 0 |  |
| Not available | 4 | 3 | 1 |  |
| TNM stage |  |  |  | 0.540 |
| I | 26 | 18 | 8 |  |
| II+ III | 3 | 3 | 0 |  |
| Not available | 4 | 3 | 1 |  |
